# Toward identifying molecules responsible for the peculiar properties of the G-layer in tension wood fibres

**DOI:** 10.1186/1753-6561-5-S7-P121

**Published:** 2011-09-13

**Authors:** Fernanda Guedes, Miyuki Takeuchi, Françoise Laurans, Gilles Pilate

**Affiliations:** 1 INRA, UR588 Amélioration, Génétique et Physiologie Forestières, F–45075 Orléans cedex 2, France

## Background

Due to its peculiar properties, tension wood formation constitutes a remarkable adaptation mechanism, that makes possible for the tree to reorientate its axes (stem and branches) in response to environnemental cues. In poplar, tension wood fibres harbour an extra cell wall layer, the G-layer, responsible for the peculiar mechanical properties of tension wood. This G-layer is very thick, most likely devoid of lignins and strongly enriched in highly cristalline cellulose. In addition, cellulose microfibril orientation is almost parallel to the fibre axis.

We aim to identify molecular actors responsible for the tensioning of cellulose microfibrils and we choose as candidate, molecules containing complex carbohydrates, such as pectin and the glycosylated part of arabinogalactan proteins. Indeed, a wide array of different carbohydrates has been recently evidenced in the G-layer, suggesting the occurrence of complex polysaccharides other than cellulose within this layer (1, 2).

## Material and methods

As a first step, we realized a comparative study between tension and opposite wood fibres using immunochemistry. A number of antibodies raised against different polysaccharide epitopes were assessed.

## Results

**Figure 1:**
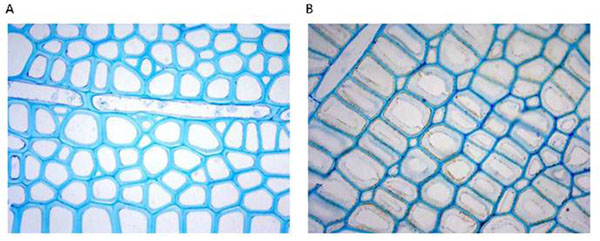
A: in opposite wood, LM5 labeling is restricted to the primary cell wall. B: strong labeling of the G-layer in mature tension wood fibres.

The study revealed important differences in the distribution of the labeling with the kind of wood, the cellular type and within a single fibre between the different cell-wall layers. When using AX1 antibody directed against arabinoxylans, the secondary cell wall layers exhibit a very strong labeling whereas G-layers were completely devoid of labeling (3). With LM5 antibodies (directed against β(1-4)galactans, opposite wood is mainly labeled at the primary wall (Figure 1A), whereas in mature tension wood the G-layer is strongly labeled (Figure 1B) as already observed by (4). With JIM14 antibody directed against cell surface arabinogalactan-proteins, a uniform but moderate labeling was visible on the middle lamella and primary cell wall of fibers, ray-cells and vessels from both opposite and tension wood. In addition, a strong labeling appears at the inner surface of the G-layer (5). The labeling of antibodies directed against the protein moiety of poplar fasciclin-like arabinogalactan proteins are also detected in the G-layer forming fibres, and mainly at the inner surface of G-layers whereas this labeling is hardly present on primary walls which were labelled with JIM14. With CCRC-M7 antibodies directed against RhamnoGalacturonan I, the labeling is restricted to the G-layer of young tension wood fibres and more specifically to the innerside of the G-layer.

## Conclusion

Our results strongly suggest the involvement of pectin and arabinogalactan proteins in the building of the G-layer.
